# Supplementary material for: Chromothripsis during telomere crisis is independent of NHEJ, and consistent with a replicative origin
Source: Genome Res. 2019 May;29(5):737–49. doi: 10.1101/gr.240705.118 (PMC6499312; doi:10.1101/gr.240705.118)
Supplement: Supplemental Material [file supp_gr.240705.118_Supplemental_file_1.zip › contigs/annotated_contigs/DB91/contig.2.DB91_length_817_mean_cov_33.3439412485.docx]

**DB91_length_817_mean_cov_33.3439412485**

```
TGGAAGATAGTCAACAAGCCAACTAATATAAACAGCTTCCTAAGCAGGGTGATATATGCATTGAGAAAGACCACAGAAGTCAGATGGGT
 >chr7:115063388-115063824 - E=2e-247
GCTGGTCAGACCTCAACCTTCCCAGTTCATCTGCTATAACTATTAAATTTTTTAACTGAGCCATAGGCTGTGTCTGAGTATTTACTTGT

TTTAGATTATTTTATTCAACCCCAAATAGAAGTTTAAATATGGTCTCATGATCCATGTAACAATATTTATATAAAAACTGGGGAGTTAG

ATAAAACCACTGAGTATATTTTGACTGCACAATTTTAATAAAATCTCATGTTTCAAAATAGAGAGTTTTTTTTTGTTGTTTGATGAACA

CCAGACATTAGCGCACTGTTCATATGCCCTTCTTTGAAAACTAGCTCATTGGAGAGGAGATTGACCATTTACGATCTCTC|GTAAC|AG
                                                                                      >
TAGACTTAAGAGATAAAAATAAATTTTACTTGCTTAGATGAAAAATATGTTAAGAAAAATTTATAGATGATCTAATAACATGTAGCTTT
chr7:113775575-113775951 + E=2e-214
TTGGTAAATATAAATTGAGACTAGCTTTGAAGTTTAAAATATATAAAGCAGTAAGTTCAGTGACCCAAGTAGTGTTATGCATTAGCTCA

AAGAGAAATCTATGATGTATTAAATCTGTTTCTGATTTTGAAAAATTTGTAGTTACAAAATCTAATTTACTTAACCCATAAAATAAAGG

CTGTGTTCATGGATTTGCTGTGCCATTCCCCATGTAATATTTGTAAAGCAAATGTGAATTTCCTTCCTAGTTCTCACCCTGAAGGAGAT

AATTCTGATAATTAGAGA
```
